# Activation of Notch1 signalling promotes multi-lineage differentiation of c-Kit^POS^/NKX2.5^POS^ bone marrow stem cells: implication in stem cell translational medicine

**DOI:** 10.1186/s13287-015-0085-2

**Published:** 2015-05-09

**Authors:** Ranran Ding, Xiaofan Jiang, Yanping Ha, Zhenliang Wang, Junli Guo, Hanguo Jiang, Shaojiang Zheng, Zhihua Shen, Wei Jie

**Affiliations:** Department of Pathology, Guangdong Medical University, Zhanjiang, 524023 China; Cardiovascular Institute of Affiliated Hospital, Hainan Medical College, Haikou, 571199 China

## Abstract

**Introduction:**

Transplantation of bone marrow mesenchymal stem cells (BMSCs) can repair injured hearts. However, whether BMSC populations contain cells with cardiac stem cell characteristics is ill-defined. We report here that Notch signalling can promote differentiation of c-Kit^POS^/NKX2.5^POS^ BMSCs into cardiomyocyte-like cells.

**Methods:**

Total BMSCs were isolated from Sprague–Dawley rat femurs and c-Kit^POS^ cells were purified. c-Kit^POS^/NKX2.5^POS^ cells were isolated by single-cell cloning, and the presence of cardiomyocyte, smooth muscle cell (SMC), and endothelial cell differentiation markers assessed by immunofluorescence staining and semi-quantitative reverse-transcription polymerase chain reaction (RT-PCR) analysis. Levels of c-Kit and Notch1–4 in total BMSCs and c-Kit^POS^/NKX2.5^POS^ BMSCs were quantitated by flow cytometry. Following infection with an adenovirus over-expressing Notch1 intracellular domain (NICD), total BMSCs and c-Kit^POS^/NKX2.5^POS^ cells were assessed for differentiation to cardiomyocyte, SMC, and endothelial cell lineages by immunofluorescence staining and real-time quantitative RT-PCR. Total BMSCs and c-Kit^POS^/NKX2.5^POS^ cells were treated with the Notch1 ligand Jagged1 and markers of cardiomyocyte, SMC, and endothelial cell differentiation were examined by immunofluorescence staining and real-time quantitative RT-PCR analysis.

**Results:**

c-Kit^POS^/NKX2.5^POS^ cells were present among total BMSC populations, and these cells did not express markers of adult cardiomyocyte, SMC, or endothelial cell lineages. c-Kit^POS^/NKX2.5^POS^ BMSCs exhibited a multi-lineage differentiation potential similar to total BMSCs. Following sorting, the c-Kit level in c-Kit^POS^/NKX2.5^POS^ BMSCs was 84.4%. Flow cytometry revealed that Notch1 was the predominant Notch receptor present in total BMSCs and c-Kit^POS^/NKX2.5^POS^ BMSCs. Total BMSCs and c-Kit^POS^/NKX2.5^POS^ BMSCs overexpressing NICD had active Notch1 signalling accompanied by differentiation into cardiomyocyte, SMC, and endothelial cell lineages. Treatment of total BMSCs and c-Kit^POS^/NKX2.5^POS^ BMSCs with exogenous Jagged1 activated Notch1 signalling and drove multi-lineage differentiation, with a tendency towards cardiac lineage differentiation in c-Kit^POS^/NKX2.5^POS^ BMSCs.

**Conclusions:**

c-Kit^POS^/NKX2.5^POS^ cells exist in total BMSC pools. Activation of Notch1 signalling contributed to multi-lineage differentiation of c-Kit^POS^/NKX2.5^POS^ BMSCs, favouring differentiation into cardiomyocytes. These findings suggest that modulation of Notch1 signalling may have potential utility in stem cell translational medicine.

**Electronic supplementary material:**

The online version of this article (doi:10.1186/s13287-015-0085-2) contains supplementary material, which is available to authorized users.

## Introduction

Stem cell transplantation is emerging as a promising method to repair heart injuries [1-3]. Stem cells are self-replicating multipotent cells that can differentiate into a variety of cell types under certain conditions. Various types of stem cells, including bone marrow cells (BMCs), mesenchymal stem cells, haematopoietic stem cells, and adipose-derived stem cells, have been used in cellular therapies to repair damage following myocardial infarction (MI). Phase I and II clinical trials have shown that transplantation of adult BMCs in patients with ischaemic heart disease improves left ventricle function and infarct size even at long-term follow-up, compared with standard therapy [4]. However, several recent clinical trials (SWISS-AMI, CELLWAVE, and C-CURE) for MI therapy involving BMCs have produced conflicting results [5-7], leading to debate concerning the efficacy of BMCs in treating heart disease [8].

The discovery of endogenous stem cells within heart tissue, termed cardiac stem cells (CSCs), offers great potential for stem cell research [9]. CSCs have self-renewal and differentiation capacities that are necessary and sufficient for MI repair [10]. The phase I clinical trials SCIPIO (ClinicalTrials.gov NCT00474461) and CADUCEUS (ClinicalTrials.gov NCT00893360) have been conducted using autologous CSCs [11,12]. The feasibility, safety, and effectiveness of autologous CSC injection were assessed in these trials, with encouraging preliminary results evidenced by a reduction in the myocardial scar mass or improvement in the left ventricular ejection fraction following cell treatment. However, a major obstacle limiting the clinical application of endogenous CSCs is the requirement for heart tissues as a cellular source, which increases the risk of injury and complications. Furthermore, obtaining the desired cell numbers for *in vivo* transplantation is time consuming because heart tissue-derived CSCs grow slowly. There is therefore a need for an alternative and easily accessible cell source that can be substituted for endogenous CSCs.

Mesenchymal stem cells are multipotent stem cells that can be easily obtained and handled, and which exhibit multilineage differentiation potential [13]. As ideal seed cells, mesenchymal stem cells have been widely used in tissue engineering, cell transplantation, and gene therapy. Mesenchymal stem cell transplantation contributes to the recovery of heart injuries, including those caused by MI, mainly through angiogenesis, paracrine signalling, activation of endogenous CSCs, and anti-inflammatory effects – but not differentiation [14]. In the C-CURE trial, bone marrow mesenchymal stem cells (BMSCs) were exposed to a cocktail of cardiogenic growth factors prior to cell transplantation, which promoted *in vivo* functions post transplantation [6]. This effect demonstrates that activating certain signalling pathways in stem cells can promote desirable biofunctions *in vivo*. The heterogeneity of BMSCs may account for their low efficiency of cardiomyocyte differentiation *in vivo*, thus limiting their long-term potential to repair heart injuries. The isolation of a specific subpopulation of MSCs with a tendency for cardiomyocyte differentiation under the influence of specific signalling would therefore be of great value to stem cell translational medicine.

The Notch signalling pathway is highly conserved evolutionarily and operates in both vertebrates and invertebrates. Notch signalling pathways are activated by interactions between adjacent cells, and influence the regulation of cell, tissue, and organ differentiation and development [15]. The Notch signalling pathway includes receptors, ligands, DNA-binding proteins, and effector molecules. There are four receptors (Notch1 to Notch4) and five ligands (DII1, DII3, DII4, Jaggedl, and Jagged2) in mammalian cells. Notch signalling occurs close to the cell surface receptor upon Notch ligand binding, with the receptor releasing a notch receptor intracellular domain (NICD) from the cell membrane. This NICD translocates to the nucleus to regulate gene transcription, thereby influencing cell proliferation and differentiation [15]. Notch signalling is involved with cell fate decisions including proliferation, lineage commitment, and terminal differentiation in many types of adult stem cells [16]. In heart tissue-derived c-kit^POS^ CSCs, activation of Notch1 signalling directly upregulates Nkx2.5 expression, promoting cardiomyocyte commitment [17]. Because Notch signalling has a remarkable cell-context dependency [18], we investigated whether Notch signalling affects c-Kit^POS^ BMSCs.

In the present study, we assessed whether populations of BMSCs included c-Kit^POS^ cells that displayed CSC differentiation properties and evaluated the effectiveness of Notch signalling activation for promoting differentiation of c-Kit^POS^ BMSCs. We identified c-Kit^POS^/NKX2.5^POS^ BMSCs in total BMSC populations, and activation of Notch1 promoted multilineage differentiation of these cells. These findings suggest that targeted modulation of Notch1 signalling may be useful in stem cell translational medicine.

## Methods

### Isolation, morphology, and functional identification of rat BMSCs

Sprague–Dawley rats (6 weeks old, both genders) were purchased from the Experimental Animal Center of Guangdong Medical University, Zhanjiang, China. All animal procedures were conducted in accordance with protocols approved by the Institutional Animal Care and Use Committee of Guangdong Medical University. Unsorted total BMSCs were isolated from rat femurs according to a previously described protocol [19,20]. Briefly, rats were sacrificed with chloroform anaesthesia, and femurs were aseptically removed and soaked in 75% ethanol for 10 minutes. Bone marrow was then flushed out using Dulbecco’s modified Eagle’s medium (DMEM; Hyclone, Beijing, China) supplemented with 2% (v/v) foetal bovine serum (FBS; Gibco, Grand Island, NY, USA). The released cells were collected, and the red blood cells in collected bone marrow-derived primary cells were removed using red blood cell lysis buffer (Sigma-Aldrich, Shanghai, China). Cell pellets were washed three times with phosphate-buffered saline (PBS) and cells were resuspended in DMEM supplemented with 10% FBS, 100 U/ml recombinant rat leukaemia inhibitory factor (LIF3010; EMD Millipore, Billerica, MA, USA), 100 U/ml penicillin, and 0.1 μg/ml streptomycin, and were maintained at 37°C in saturated humidity with 5% carbon dioxide. After 2 days, nonadherent cells were removed, and medium changes were performed every 3 days thereafter. Cell morphology was monitored under an inverted microscope. Fourth passage cells were used for differentiation experiments. Total BMSCs (3 × 10^3^ cells/cm^2^) were seeded in six-well plates. For osteogenesis, cells were treated with inducing medium (RASMX-90021; Cyagen Biosciences, Guangzhou, China) for 3 weeks. Cells were then fixed with 4% formaldehyde for 30 minutes and stained with Alizarin Red. For adipocyte differentiation, cells were maintained in adipocyte genesis medium (RASMX-90031; Cyagen Biosciences) for 3 weeks. Cells were then stained with Oil Red O [21]. For smooth muscle cell (SMC) differentiation, cells grown on coverslips were treated with medium containing 10 ng/ml transforming growth factor-β1 (100–21; PeproTech, Rocky Hill, NJ, USA) for 8 days and then subjected to immunofluorescence staining for the SMC marker smooth muscle myosin heavy chain.

### Isolation, cloning, morphology, and identification of c-Kit^POS^/NKX2.5^POS^ BMSCs

Third-passage BMSCs were harvested and c-Kit^POS^ cells isolated using a magnetic activated cell sorting (MACS) system (Miltenyi Biotec, Bergisch Gladbach, Germany) as described previously [22,23]. Diluted c-Kit^POS^ cells were seeded in six-well plates and maintained in DMEM supplemented with 10% FBS, 0.2 mmol/l glutathione (G5763; Sigma Aldrich), 2.5 U/ml erythropoietin (287-TC-500; R&D Systems, Minneapolis, MN, USA), and 10 ng/ml recombinant fibroblast growth factor (400–29; PeproTech). Cellular morphology of single cell-derived clones was monitored at 3, 7, 14, 21, and 28 days, and confluent cells were used to assess marker expression by immunofluorescence staining and RT-PCR analysis. Cells that were positive for c-Kit and early cardiac marker NKX2.5, but negative for markers of mature cardiomyocytes (α-sarcomeric actin, cardiac troponin T (cTnT)), SMCs (SM22α), and endothelial cells (von Willebrand factor (vWF)), were used in subsequent experiments. These cells were classed as c-Kit^POS^/NKX2.5^POS^ BMSCs. Differentiation of c-Kit^POS^/NKX2.5^POS^ BMSCs into osteocytes, adipocytes, and SMCs was monitored as per the total BMSCs described above.

### NICD overexpression by adenovirus-mediated transduction

NICD cDNA (5,468 to 7,820 nucleotides; NM_001105721) was obtained from the cDNA library of Genechem (Shanghai, China) with the following primers: 5′-AGG TCG ACT CTA GAG GAT CCC GCC ACC ATG CGC AAG CGC AGG CGG CA-3′ and 5′-TCC TTG TAG TCC ATA CCC TTA AAT GCC TCT GGA ATG TGG G-3′. The 2,399 base pair PCR product was cloned into a linearised adenovirus plasmid GV314 (Genechem) with T4 DNA ligase and transfected into competent *Escherichia coli* cells. Positive clones were selected by ampicillin resistance and then sequenced by ABI3730 sequencing analysis (Invitrogen, Shanghai, China). The NICD overexpression adenovirus (NICD-Ad) was packaged in HEK293T cells and purified with an Adeno-X™ Virus Purification Kit (BD Biosciences, San Jose, CA, USA). The endpoint dilution method was used to determine the viral titre. Adenovirus particles expressing a scrambled sequence (NC-Ad; purchased from Genechem) served as a negative control.

For cell infection, total BMSCs and c-Kit^POS^/NKX2.5^POS^ BMSCs were seeded in six-well plates with or without coverslips. After 24 hours, the NICD-Ad or NC-Ad virus was added to the cells at a multiplicity of infection of 100 in the presence of 4 μg/ml polybrene. Cells not transfected with virus were classified MOCK. Twenty-four hours after infection, medium was changed to complete DMEM and then changed every 3 days. After 72 hours, the viral infection efficiency was assessed under fluorescence microscopy. Cells were harvested for experiments at day 8 after infection.

### Determination of Notch receptors and c-Kit expression by flow cytometry

Total BMSCs and c-Kit^POS^/NKX2.5^POS^ BMSCs (2 × 10^6^ cells) were fixed in cold 70% ethanol overnight. After washing with PBS, cells were incubated with primary antibodies against Notch1 to Notch4 and c-Kit at room temperature for 1 hour. Following three washes with PBS, cells were incubated with fluorescein isothiocyanate-conjugated or phycoerythrin-conjugated IgG in the dark at room temperature for 30 minutes. Samples were then subjected to flow cytometric analysis using a BD FACSCanto II (BD Biosciences). Data were analysed with FACSDiva software (BD Biosciences).

### Exogenous Jagged-1-induced total BMSC and c-Kit^POS^/NKX2.5^POS^ BMSC differentiation

Total BMSCs and c-Kit^POS^/NKX2.5^POS^ BMSCs were maintained in complete DMEM and seeded in six-well plates with coverslips or 6 cm dishes. For induced differentiation, five treatment groups were established and cultured in DMEM + 10% FBS: Jagged1, with 2.5 μg/ml Jagged1 Fc Chimera (99-JG; R&D Systems); IgG, with 2.5 μg/ml IgG (14131; Sigma Aldrich); Notch1 inhibitor *N*-(2S-(3,5-difluorophenyl)acetyl)-l-alanyl-2-phenyl-1,1-dimethylethyl ester-glycine (DAPT), with 2.5 μg/ml Jagged1 Fc Chimera and 10 μmol/ml DAPT (D5942; Sigma Aldrich) dissolved in dimethyl sulfoxide; DMSO, with ≤0.03% dimethyl sulfoxide (v/v); and MOCK, with medium alone. Medium was changed every 3 days. After 8 days, cells grown on coverslips were subjected to immunofluorescence staining, while cells grown in dishes were subjected to mRNA expression analysis.

### RNA isolation, reverse transcription, and PCR

Total BMSCs and c-Kit^POS^/NKX2.5^POS^ BMSCs were subjected to mRNA expression analysis. Total RNA was extracted with RNAiso Plus (D9108B; TaKaRa, Dalian, China). To generate cDNA, total RNA (1 μg) was reverse transcribed using an RT kit (DRR047A; TaKaRa) with an oligo(dT18) primer. Quantitative PCR was conducted using a LightCycler480 II instrument (Roche China, Guangzhou, China), and semi-quantitative PCR was performed using a Mastercycler® Gradient Thermal cycler (Eppendorf, Wesseling-Berzdorf, Germany). Primers were synthesised by Sangon Biotech (Shanghai, China) and are listed in Additional file 1. For quantitative PCR, reaction volumes of 20 μl included 10 μl SYBR® Premix Ex Taq™ (catalogue number DRR820A; Takara), 0.8 μl each primer (10 μM), 2 μl cDNA, and 6.4 μl ddH_2_O. PCR amplification conditions were as follows: denaturation at 95°C for 30 seconds, followed by 45 cycles of 95°C for 5 seconds, and 60°C for 20 seconds. The relative amount of target mRNAs was determined using cycle threshold values and plotted as the fold change compared with control groups (2^−∆∆CT^ method). Semi-quantitative PCR conditions were 95°C for 5 minutes, followed by 31 cycles at 95°C for 45 seconds, 58°C for 45 seconds, and 72°C for 1 minute. PCR products were separated by 1.5% agarose gel electrophoresis and visualised under ultraviolet light using a gel documentation system (Bio-Rad, Hercules, CA, USA). For all PCR analyses, the expression level of β-actin served as a loading control.

### Immunofluorescence staining

Cells grown on coverslips were subjected to indirect immunofluorescence to identify the expression and location of cleaved Notch1 (NICD) and differentiation markers. Following overnight incubation with primary antibodies at 4°C, cells were washed three times with PBS. Cells were then incubated with fluorescein isothiocyanate-conjugated or phycoerythrin-conjugated IgG (H + L; Proteintech, Wuhan, China) at room temperature for 30 minutes. Nuclei were counterstained with 4′,6-diamidino-2-phenylindole (5 μg/ml) at room temperature for 15 minutes. Images were captured under a laser scanning confocal microscope (TCS SP5 II; Leica, Wetzlar, Germany). Primary antibodies used are listed in Additional file 2.

### Statistical analysis

Statistical analyses were performed using PRISM Software (Version 5; GraphPad Software, San Diego, CA, USA). Data are expressed as the mean ± standard deviation. Student’s *t* test was used to analyse differences between two groups. For multiple group comparisons, analysis of variance was carried out followed by the Student–Newman–Keuls test. *P* <0.05 was considered statistically significant.

## Results

### Isolation of c-Kit^POS^/NKX2.5^POS^ cells from total BMSCs and assessment of cellular morphology

**Figure 1:**
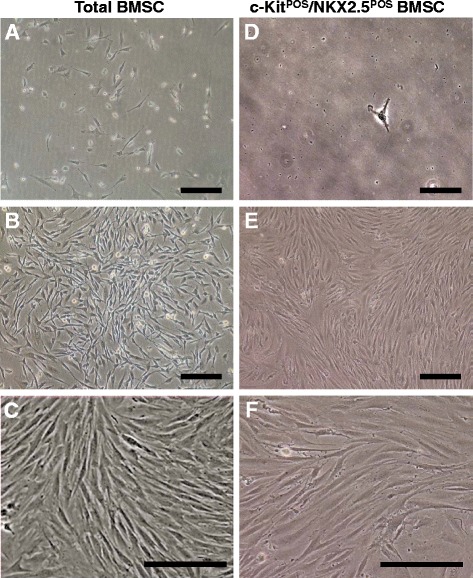
Representative morphology of total and c-Kit^POS^/NKX2.5^POS^ bone marrow mesenchymal stem cells. **(A)** Bone marrow mesenchymal stem cells (BMSCs) gradually showed fibroblast-like shapes by day 3 after plating. **(B)** Cells reached confluency at day 14. **(C)** High magnification of confluent BMSCs. **(D)** A single c-Kit^POS^/NKX2.5^POS^ BMSC showing a polygonal shape at 3 days after plating. **(E)** The cell clone at confluency after 21 days. **(F)** High magnification of confluent c-Kit^POS^/NKX2.5^POS^ BMSCs. Scale bar: (A, B, D, E) 30 μm, and (C, F) 60 μm. Original magnifications: (A, B, D, E) ×100, and (C, F) ×200.

**Figure 2:**
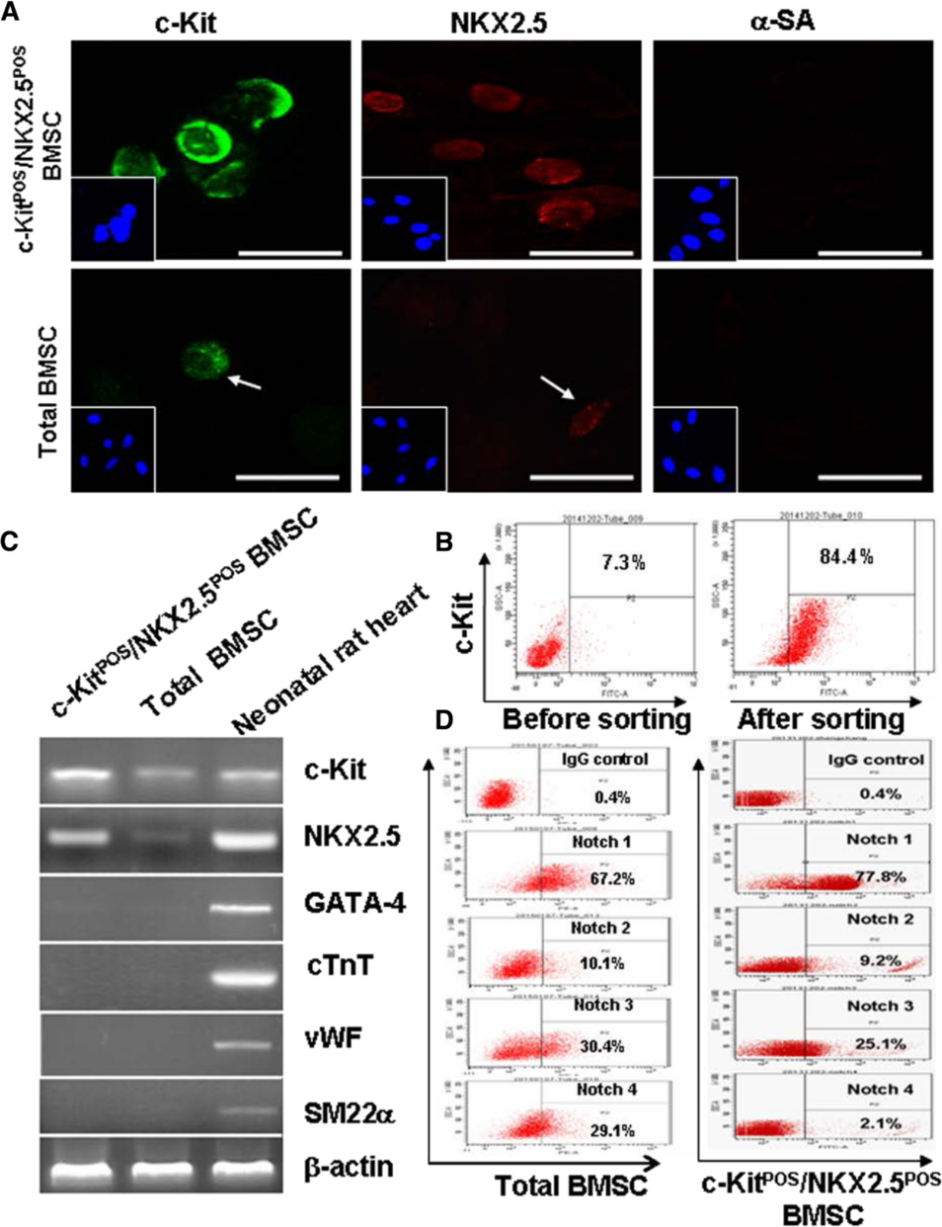
Phenotypic identification of c-Kit^POS^/NKX2.5^POS^ bone marrow mesenchymal stem cells. **(A)** Confluent, c-Kit^POS^/NKX2.5^POS^ bone marrow mesenchymal stem cells (BMSCs) were passaged and third-passage cells were subjected to immunofluorescence staining for c-Kit, NKX2.5, and α-sarcomeric actin (α-SA). Unsorted total BMSCs served as control. **(B)** Assessment of c-Kit expression in total BMSCs before and after magnetic activated cell sorting by flow cytometric analysis. **(C)** Semi-quantitative RT-PCR analysis of markers for stem cells (c-Kit), cardiomyocytes (NKX2.5, GATA-4, and cardiac troponin T (cTnT)), smooth muscle cells ( SM22α) and endothelial cells (von Willebrand factor (vWF)). A sample from a neonatal Sprague–Dawley rat heart was used as positive control. **(D)** Flow cytometry was performed to detect the presence of Notch1 to Notch4 receptors. For immunofluorescence staining, target proteins were detected with fluorescein isothiocyanate (FITC)-conjugated or phycoerythrin (PE)-conjugated IgG. Images were captured by fluorescence microscopy. Scale bar: 50 μm. For semi-quantitative RT-PCR analysis, β-actin mRNA was used as an internal control. For flow cytometric analysis, isotype control IgG was used to set the threshold.

Total BMSCs are a heterogeneous cell population including various phenotypical cell subpopulations [24]. We successfully isolated BMSCs from rat femurs according to a protocol described previously [19,20]. Unsorted total BMSCs displayed a typical fibroblast-like morphology (Figure 1A,B,C). c-Kit is a receptor tyrosine kinase which is bound by stem cell factor to activate signalling pathways crucial for stem cell functions such as heart tissue-derived CSC migration [25]. Furthermore, c-Kit is a relatively stable stem cell marker [26] which can be reliably detected in heart tissue-derived CSCs even after 40 passages [27]. We employed MACS to isolate c-Kit^POS^ cells from total BMSCs. Single-cell cloning was then used to establish c-Kit^POS^ and NKX2.5^POS^ cells that were negative for GATA-4, cTnT, SM22α, and vWF expression, as determined by immunofluorescence staining and semi-quantitative RT-PCR (Figure 2A,C). These cells were defined as c-Kit^POS^/NKX2.5^POS^ BMSCs, and single c-Kit^POS^/NKX2.5^POS^ BMSCs could form clones well (see Additional file 3). c-Kit^POS^/NKX2.5^POS^ BMSCs exhibited a fibroblast-like morphology similar to that of total BMSCs, and there were no obvious morphological differences between the two cell types (Figure 1). However, c-Kit^POS^/NKX2.5^POS^ BMSCs grew slower than the total BMSCs, but faster than heart tissue-derived CSCs (see Additional file 4).

### c-Kit and Notch receptor expression by total BMSCs and c-Kit^POS^/NKX2.5^POS^ BMSCs

We performed flow cytometry to analyse the expression of c-Kit and Notch1 to Notch4 receptors on total BMSCs and c-Kit^POS^/NKX2.5^POS^ BMSCs. Before MACS sorting, 7.3% of total BMSCs were c-Kit-positive, while 84.4% of cells were c-Kit-positive following a single round of sorting (Figure 2B). This result indicates that MACS is a suitable method for enriching c-Kit-positive cell populations from BMSCs. Varied expression levels of Notch receptors were detected in total BMSCs and c-Kit^POS^/NKX2.5^POS^ BMSCs (Figure 2D). Notch1 was the predominantly expressed form of receptor in both total BMSC and c-Kit^POS^/NKX2.5^POS^ BMSC populations, Following sorting, Notch1-positive cells increased from 67.2% in total BMSCs to 77.8% in c-Kit^POS^/NKX2.5^POS^ BMSCs (Figure 2D). These results suggest that Notch1 signalling plays a crucial role in the biology of c-Kit^POS^/NKX2.5^POS^ BMSCs, which supports our rationale for investigating Notch1.

### Forced Notch1-NICD expression leads to Notch1 signalling activation and multilineage differentiation in total BMSCs and c-Kit^POS^/NKX2.5^POS^ BMSCs

**Figure 3:**
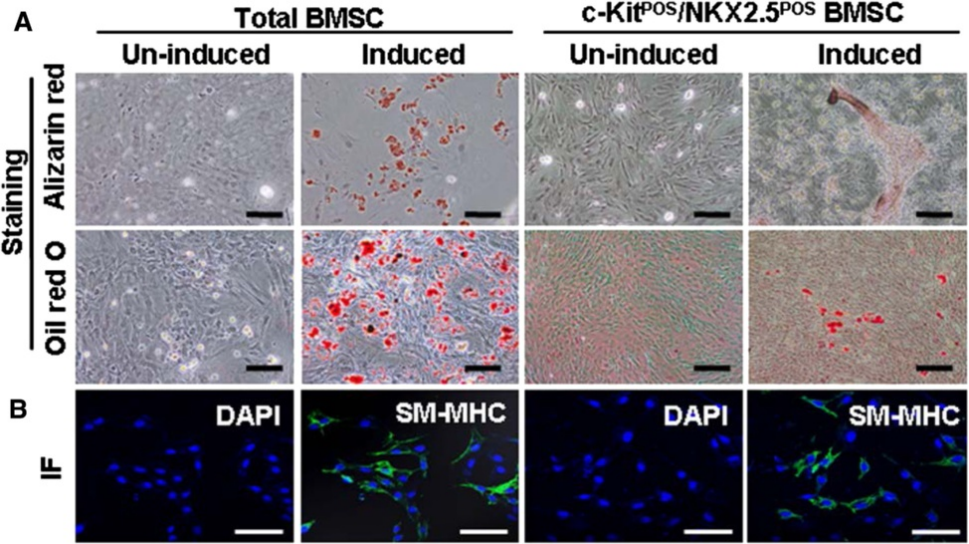
Functional identification of total and c-Kit^POS^/NKX2.5^POS^ bone marrow mesenchymal stem cells. **(A)** Uninduced and induced bone marrow mesenchymal stem cells (BMSCs) and c-Kit^POS^/NKX2.5^POS^ BMSCs were stained after 21 days of osteogenic or adipogenic differentiation with Alizarin Red and Oil Red O, respectively. **(B)** For smooth muscle cell differentiation, BMSCs were treated with transforming growth factor-β1-supplemented medium for 8 days, and then subjected to immunofluorescence (IF) staining for smooth muscle myosin heavy chain (SM-MHC). Target proteins were detected with fluorescein isothiocyanate-conjugated IgG. Nuclei were counterstained with 4′,6-diamidino-2-phenylindole (DAPI). Original magnification: (A) ×200. Scale bar: 30 μm.

Total BMSCs and c-Kit^POS^/NKX2.5^POS^ BMSCs were treated with specific agents to induce osteogenic, adipogenic, and SMC differentiation, and differentiated cells expressed typical markers of differentiation (Figure 3). Both total BMSCs and c-Kit^POS^/NKX2.5^POS^ BMSCs therefore displayed multilineage differentiation potential, suggesting that these cells could be induced to differentiate into desired cell types by other cues.

**Figure 4:**
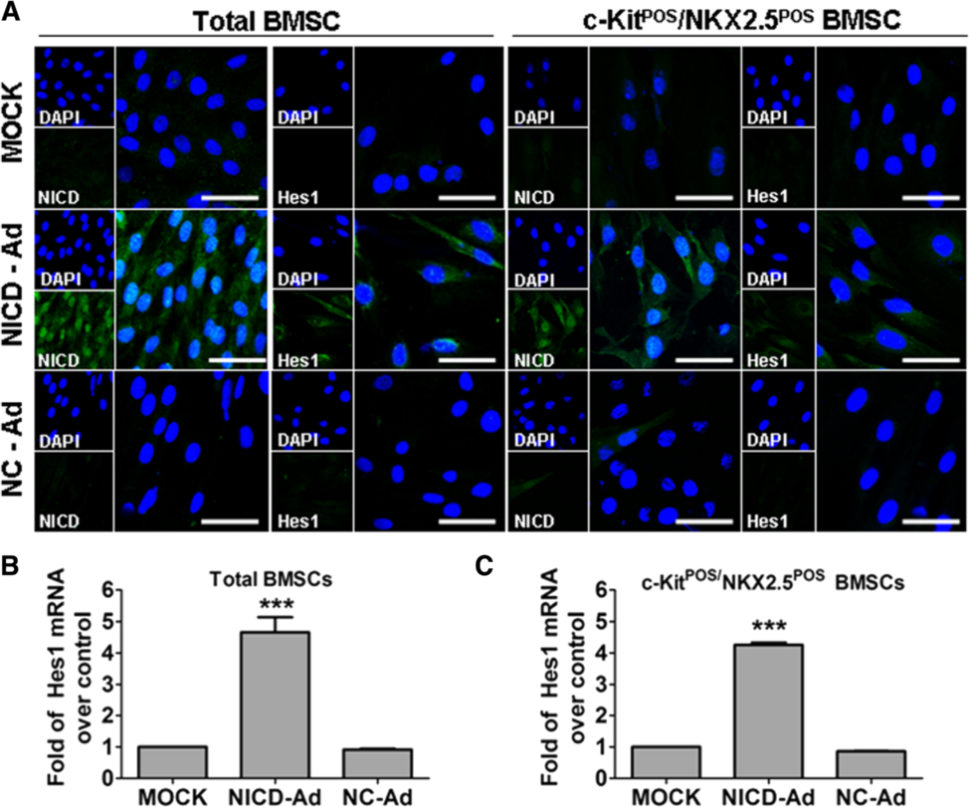
Forced NICD expression activation of Notch1 signalling in total and c-Kit^POS^/NKX2.5^POS^ bone marrow mesenchymal stem cells. Total bone marrow mesenchymal stem cells (BMSCs) and c-Kit^POS^/NKX2.5^POS^ BMSCs were infected with NICD-Ad or NC-Ad, with uninfected cells serving as an additional control (MOCK). After 8 days, the cells were harvested for **(A)** immunofluorescence staining for notch receptor intracellular domain (NICD) and Hes1, and **(B)** real-time quantitative RT-PCR analysis of Hes1 mRNA expression. For immunofluorescence staining, target proteins were detected with fluorescein isothiocyanate-conjugated IgG. **(C)** Nuclei were counterstained with 4′,6-diamidino-2-phenylindole (DAPI). Images were captured by confocal microscopy and merged. Scale bar: 50 μm. Triplicate experiments were performed for real-time quantitative RT-PCR analysis (*n* = 3). ***P <0.001 versus other groups. NC-Ad, negative control-expressing adenovirus; NICD-Ad, NICD-expressing adenovirus.

Binding of ligand Jagged1 to the Notch1 receptor releases NICD to the nucleus. NICD forms a protein complex with CSL(CBF1/Su(H)/Lag-1), which then turns the co-repressor into a co-activator, subsequently activating transcription of target genes [15]. We constructed an adenovirus to overexpress NICD (NICD-Ad) independent of Notch1 activation. At 8 days post infection with NICD-Ad, we observed enhanced Hes1 mRNA expression in both total BMSCs and c-Kit^POS^/NKX2.5^POS^ BMSCs, compared with expression in controls (NC-Ad and MOCK; Figure 4). These results suggest that forced expression of Notch1-NICD in total BMSCs and c-Kit^POS^/NKX2.5^POS^ BMSCs led to activation of Notch1 signalling.

**Figure 5:**
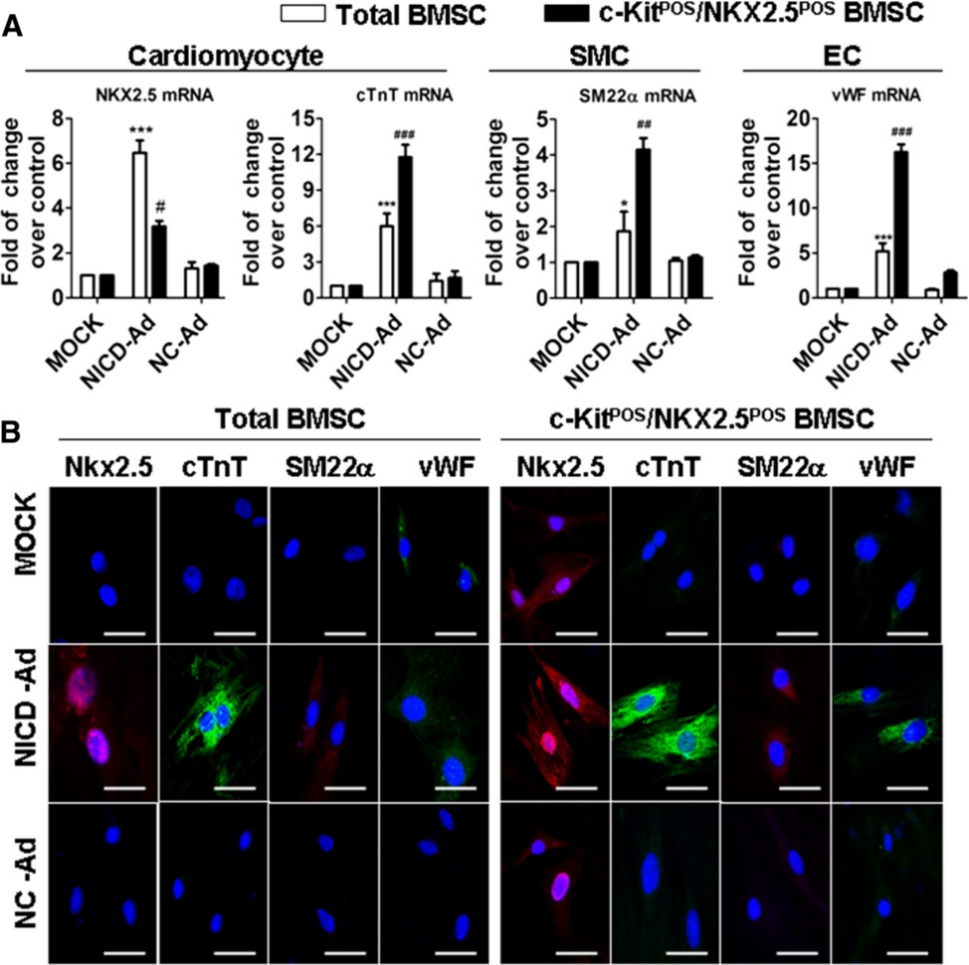
Forced NICD expression induces multilineage differentiation of total and c-Kit^POS^/NKX2.5^POS^ bone marrow mesenchymal stem cells. Total bone marrow mesenchymal stem cells (BMSCs) and c-Kit^POS^/NKX2.5^POS^ BMSCs were infected with NICD-Ad or NC-Ad, with uninfected cells serving as an additional control (MOCK). After 8 days, the cells were harvested for **(A)** real-time quantitative RT-PCR analysis and **(B)** immunofluorescence staining for markers for cardiomyocytes (NKX2.5 and cardiac troponin T (cTnT)), smooth muscle cells (SM22α), and endothelial cells (von Willebrand factor (vWF)). Triplicate experiments were performed for real-time quantitative RT-PCR analysis (*n* = 3). *P <0.05, ***P <0.001, ^#^ *P* <0.05, ^##^ *P* <0.01, ^###^ *P* <0.001 versus other associated groups. For immunofluorescence staining, images were captured by confocal microscopy. Target proteins were detected with phycoerythrin-conjugated or fluorescein isothiocyanate-conjugated IgG. Nuclei were counterstained with 4′,6-diamidino-2-phenylindole (DAPI). Scale bar: 50 μm. EC, endothelial cell; NC-Ad, negative control-expressing adenovirus; NICD, notch receptor intracellular domain; NICD-Ad, NICD-expressing adenovirus; SMC, smooth muscle cell.

We next evaluated the expression of markers of cardiomyocyte (NKX2.5 and cTnT), SMC (SM22α), and endothelial cell (vWF) differentiation. Protein and mRNA levels of NKX2.5, cTnT, SM22α, and vWF were upregulated in NICD-overexpressing total BMSCs and c-Kit^POS^/NKX2.5^POS^ BMSCs (Figure 5). These data indicate that forced expression of NICD in total BMSCs and c-Kit^POS^/NKX2.5^POS^ BMSCs activated Notch1 signalling and induced multilineage differentiation, supporting a role for Notch1 signalling in the differentiation of BMSCs. Interestingly, we also found that forced activation of Notch1 in c-Kit^POS^/NKX2.5^POS^ BMSCs led to enhanced osteogenic and adipogenic differentiation (see Additional file 5). This probably reflects the complexity of Notch1 signalling in stem cell differentiation.

### Jagged1-triggered Notch1 signalling activation induces multi-lineage differentiation

**Figure 6:**
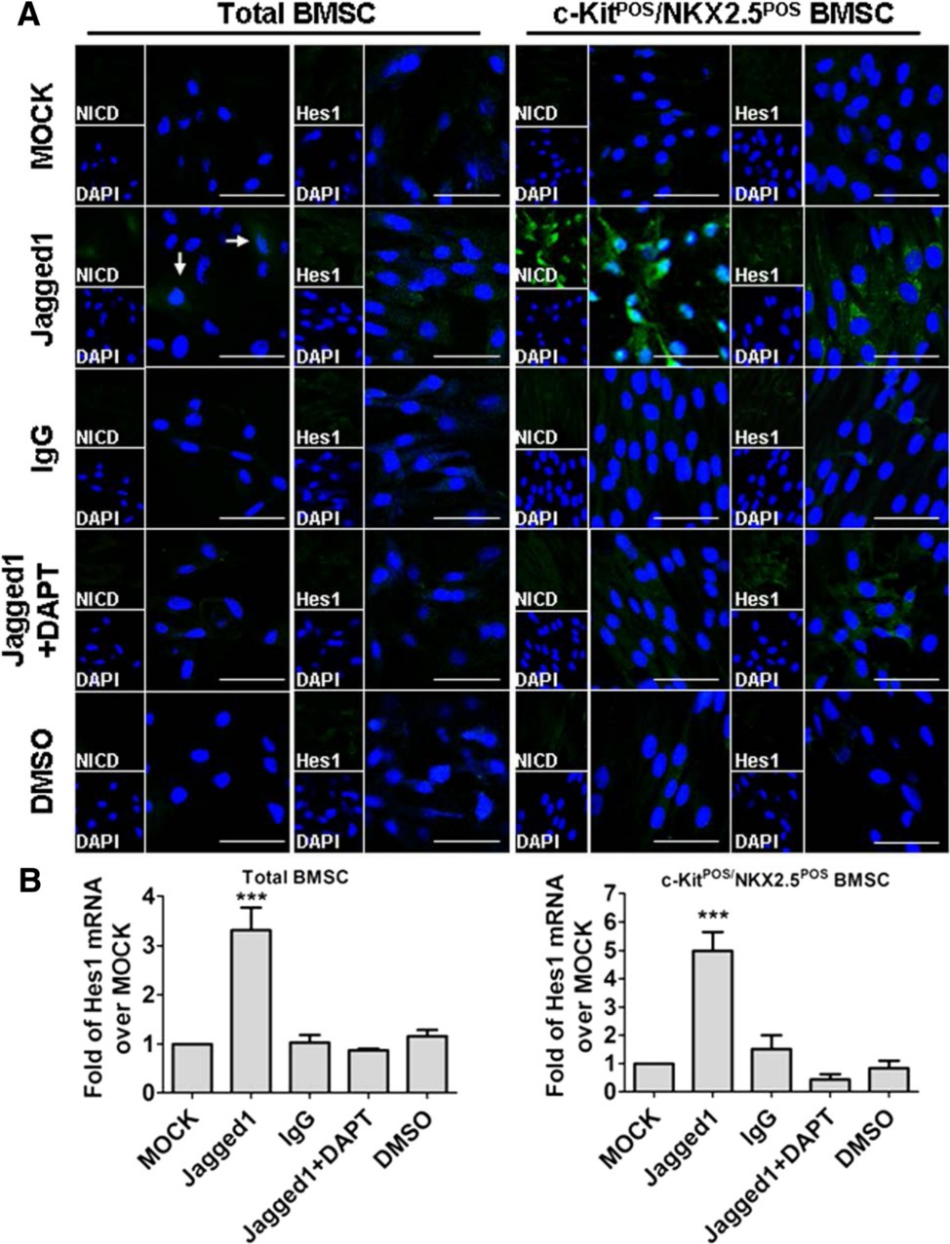
Exogenous Jagged1 activation of Notch1 signalling in total and c-Kit^POS^/NKX2.5^POS^ bone marrow mesenchymal stem cells. Total bone marrow mesenchymal stem cells (BMSCs) and c-Kit^POS^/NKX2.5^POS^ BMSCs were classified into Jagged1, IgG, *N*-(2S-(3,5-difluorophenyl)acetyl)-l-alanyl-2-phenyl-1,1-dimethylethyl ester-glycine (DAPT), dimethyl sulfoxide (DMSO), and MOCK treatment groups (see Methods). After 8 days, activation of Notch1 signalling was assessed by immunofluorescence staining of notch receptor intracellular domain (NICD) and Hes1 protein expression **(A)** and quantitative RT-PCR analysis of Hes1 mRNA expression **(B)**. For immunofluorescence staining, target proteins were detected with fluorescein isothiocyanate-conjugated IgG. Nuclei were counterstained with 4′,6-diamidino-2-phenylindole (DAPI). Scale bar: 50 μm. Representative images were captured by confocal microscopy. Triplicate experiments were performed for real-time quantitative RT-PCR analysis (*n* = 3). ***P <0.001 versus other groups.

We treated total BMSCs and c-Kit^POS^/NKX2.5^POS^ BMSCs with Jagged1 to activate Notch1 signalling, as Notch1 was the predominant Notch receptor expressed by these cells. Exogenous recombinant Jagged1 activated Notch1 signalling in both total BMSCs and c-Kit^POS^/NKX2.5^POS^ BMSCs as evidenced by upregulation of Hes1 and NICD, while the Notch1 inhibitor DAPT attenuated this effect (Figure 6). Jagged1-induced Notch1 activation in c-Kit^POS^/NKX2.5^POS^ BMSCs was stronger than that in total BMSCs, as indicated by increased immunofluorescence signals and Hes1 levels in Jagged1-treated cells (Figure 6).

**Figure 7:**
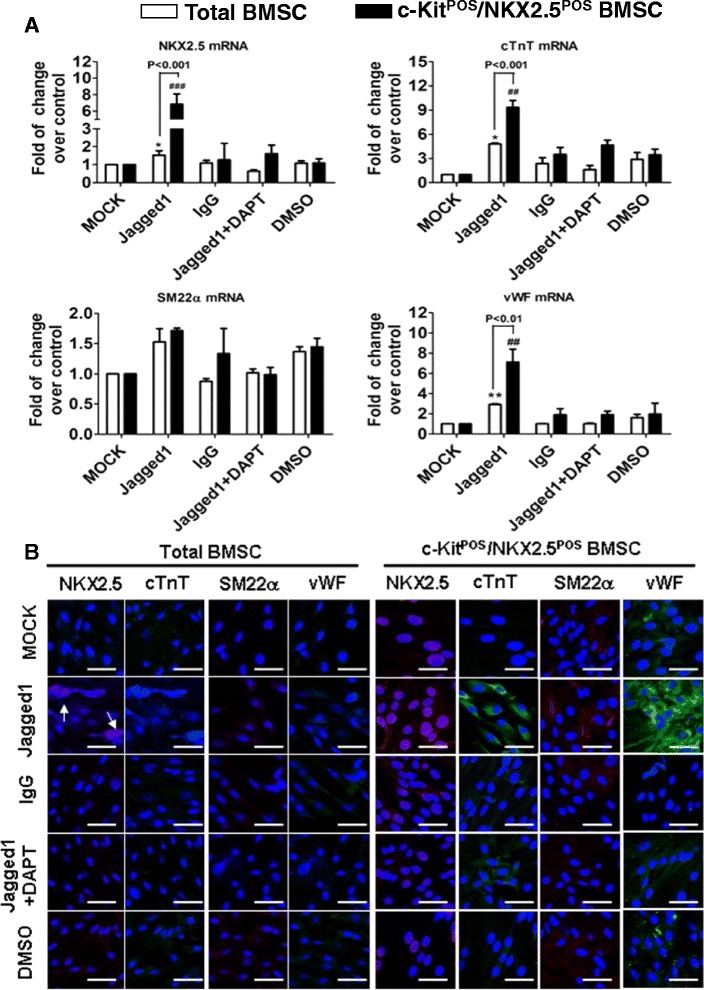
Jagged1 activation of Notch1 signalling induces multilineage differentiation of total and c-Kit^POS^/NKX2.5^POS^ bone marrow mesenchymal stem cells. Total bone marrow mesenchymal stem cells (BMSCs) and c-Kit^POS^/NKX2.5^POS^ BMSCs were classified into Jagged1, IgG, *N*-(2S-(3,5-difluorophenyl)acetyl)-l-alanyl-2-phenyl-1,1-dimethylethyl ester-glycine (DAPT), dimethyl sulfoxide (DMSO), and MOCK treatment groups (see Methods). After 8 days, the expression of differentiation markers for cardiomyocytes (NKX2.5 and cardiac troponin T (cTnT)), smooth muscle cells (SM22α), and endothelial cells (von Willebrand factor (vWF)) were examined by **(A)** real-time quantitative RT-PCR and **(B)** immunofluorescence staining. Triplicate experiments were performed for real-time quantitative RT-PCR analysis (*n* = 3). *P <0.05, **P <0.01, ^##^ *P* <0.01, ^###^ *P* <0.001 versus other associated groups. For immunofluorescence staining, target proteins were detected with phycoerythrin-conjugated or fluorescein isothiocyanate-conjugated IgG. Nuclei were counterstained with 4′,6-diamidino-2-phenylindole (DAPI). Representative images were captured by confocal microscopy. Scale bar: 50 μm.

We next assessed the differentiation of total BMSCs and c-Kit^POS^/NKX2.5^POS^ BMSCs exposed to Jagged1. Jagged1-induced Notch1 signalling led to enhanced NKX2.5, cTnT, SM22α, and vWF expression, while inhibition of Notch1 signalling by DAPT reduced their expression at both mRNA and protein levels (Figure 7). These findings demonstrate that Jagged1-triggered Notch1 signalling induced differentiation of total BMSCs and c-Kit^POS^/NKX2.5^POS^ BMSCs into cardiomyocyte, SMC, and endothelial cell lineages.

### Jagged1-activated Notch1 signalling predominantly induces cardiomyocyte differentiation in c-Kit^POS^/NKX2.5^POS^ BMSCs

**Figure 8:**
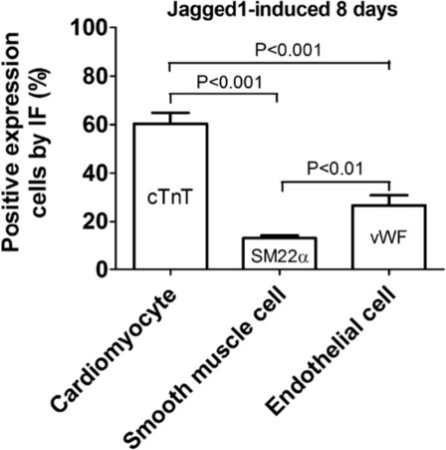
Activation of Notch1 signalling favours cardiac differentiation of c-Kit^POS^/NKX2.5^POS^ bone marrow mesenchymal stem cells. Positive immunofluorescence (IF) staining was assessed for cardiac troponin T (cTnT), smooth muscle cells (SM22α), and von Willebrand factor (vWF) in 10 representative fields of Jagged1-treated c-Kit^POS^/NKX2.5^POS^ bone marrow mesenchymal stem cells. Ratios to 4′,6-diamidino-2-phenylindole-positive cells represent differentiation potentials for cardiomyocytes, smooth muscle cells, and endothelial cells.

Following exposure of c-Kit^POS^/NKX2.5^POS^ BMSCs to exogenous Jagged1, the increases in expression of cardiomyocyte and endothelial cell lineage markers were much stronger than those in total BMSCs (Figure 7A). There was no obvious difference in expression of the SMC differentiation marker SM22α (Figure 7A). These findings suggest that c-Kit^POS^/NKX2.5^POS^ BMSCs are more responsive to Notch1 signalling stimuli than total BMSCs. We further evaluated the tendency of Jagged1-treated c-Kit^POS^/NKX2.5^POS^ BMSCs to differentiate into cardiomyocytes, SMCs, and endothelial cells by counting cTnT-positive, SM22α-positive, and vWF-positive cells in 10 representative fields of view to determine the percentages of positive cells in each lineage. Jagged1-induced activation of Notch1 signalling in c-Kit^POS^/NKX2.5^POS^ BMSCs facilitated predominately cardiomyocyte differentiation, while differentiation into endothelial and SMC lineages was much weaker (Figure 8).

## Discussion

Stem cell therapy is emerging as a promising treatment strategy for MI. Current investigations are endeavouring to identify the most suitable type of stem cell for MI treatment [28,29]. The feasibility, safety, and effectiveness of stem cell transplantation based on heart tissue-derived CSCs have been demonstrated in animal models of MI. Recent results of several phase I clinical trials have also have provided encouraging results [11,12]. However, the SWISS-AMI trial has generated conflicting results [5]. A potential explanation for the inconsistent results of these clinical trials may be the variability of cell types used [8]. Ellison and colleagues have highlighted the necessity of heart tissue-derived CSCs for MI repair [10]. However, considering the potential for damage caused by the acquisition of heart tissue for CSCs, there is a need for an alternative cell source to substitute for heart tissue-derived CSCs.

Compared with heart tissue-derived CSCs, BMSCs are able to be handled and expanded more easily [14]. Furthermore, their phenotype is not influenced by confluency during culture expansion [30]. Additionally, a meta-analysis concluded that BMSCs have promising prospects for MI treatment [31]. BMSCs exhibit a multilineage *in vitro* differentiation potential, including cardiomyocytes. However, the functions of BMSCs *in vivo* are mainly mediated in a paracrine manner [32] and these cells do not directly differentiate into functional cardiomyocytes [33]. A possible reason for this indirect effect may be the multiple subpopulations of cells with different differentiation potentials that constitute total BMSCs. Screening purified BMSCs for highly-efficient differentiation to cardiomyocytes could therefore be of great benefit to stem cell-based cardiac repair.

Because Notch signalling plays a critical role in stem cell differentiation into cardiomyocytes [17,34,35], we examined whether forced activation of Notch1 signalling could efficiently induce cardiac differentiation of BMSCs. Therefore, we infected unsorted total BMSCs with a recombinant NICD-expressing adenovirus. Forced expression of NICD in BMSCs promoted multilineage differentiation, including to cardiomyocytes, suggesting that activation of the Notch1 signalling pathway is functionally relevant in BMSC differentiation.

We next examined the function of Notch1 signalling on the c-Kit^POS^/NKX2.5^POS^ subtype of BMSCs. We successfully isolated c-Kit^POS^ cells from total BMSCs using MACS as described previously [22,23,25]. Cell clones positive for NKX2.5 were then successfully derived from single c-Kit^POS^ cells. Finally, c-Kit^POS^/NKX2.5^POS^ cells were found to be negative for markers of adult cardiomyocyte (α-sarcomeric actin, cTnT), SMC (SM22α), and endothelial cell (vWF) differentiation. The purity of c-Kit^POS^/NKX2.5^POS^ BMSCs after a single MACS reached 84.4%, significantly higher than that of the 7.3% in total BMSCs before sorting.

The use of c-Kit^POS^ BMSCs in MI treatment has been proposed by previous reports [36,37]; however, we further investigated the c-Kit^POS^/NKX2.5^POS^ subpopulation and showed that, of the four known Notch receptors, these cells predominantly expressed Notch1. This observation led us to examine the effects of ligand-induced activation of Notch1 signalling on differentiation of c-Kit^POS^/NKX2.5^POS^ BMSCs. c-Kit^POS^/NKX2.5^POS^ BMSCs proliferated slower than total BMSCs, but faster than c-Kit^POS^ CSCs derived from rat hearts, indicating that c-Kit^POS^/NKX2.5^POS^ BMSCs can be expanded more easily than heart tissue-derived c-Kit^POS^ CSCs.

Notch signalling contributes to stem cell quiescence, proliferation, and differentiation depending on the context in which the signal is activated [38]. Boni and colleagues reported that Notchl directly regulates NKX2.5 promoter activity in mouse heart-derived CSCs, thereby positively regulating cardiomyocyte differentiation [17]. Chen and colleagues demonstrated that activation of Notch signalling in mouse cardiosphere cells – a heterogeneous progenitor cell population – promotes SMC differentiation through a J kappa-recombining binding protein-dependent signalling pathway *in vitro* [39]. However, activation of Notch signalling in embryonic stem cells has been reported to produce opposite results [40]. A recent study by Matsuda and colleagues proposed that the cell density of heart tissue-derived CSC cultures influences Notch1 activation status and differentiation potential, influencing *in vivo* CSC function and potential utility for MI therapy [41]. These inconsistencies highlight the need for further efforts to clarify the exact role of Notch1 signalling in c-Kit^POS^/NKX2.5^POS^ BMSC differentiation.

We found that Notch1 was the predominant Notch receptor form expressed by both total BMSCs and c-Kit^POS^/NKX2.5^POS^ BMSCs. We therefore treated these two types of cells with exogenous Jagged1 to mimic the binding of ligand and receptor. As shown in our pilot investigation, 2.5 μg/ml exogenous Jagged1 maximised Hes1 mRNA expression (see Additional file 6); we therefore treated the cells with 2.5 μg/ml Jagged1 in the subsequent experiments. Jagged1 activated Notch1 signalling and promoted differentiation of both total BMSCs and c-Kit^POS^/NKX2.5^POS^ BMSCs into cardiomyocyte, SMC, and endothelial cell lineages. Furthermore, expression levels of cardiomyocyte and endothelial cell lineage markers in cells derived from c-Kit^POS^/NKX2.5^POS^ BMSCs were greater than those of cells derived from total BMSCs. Moreover, differentiation of the cardiomyocyte lineage was favoured over differentiation of endothelial cell and SMC lineages in c-Kit^POS^/NKX2.5^POS^ BMSCs. This suggests that c-Kit^POS^/NKX2.5^POS^ BMSCs preferentially differentiate into cardiac myocytes following activation of Notch1 signalling. These *in vitro* results support a previous report which found that Notch1 signalling in BM-derived cells is critical for cardiac repair [42]. Activation of Notch1 signalling therefore promotes c-Kit^POS^/NKX2.5^POS^ BMSC differentiation, suggesting that targeting Notch1 signalling may be beneficial for stem cell translational medicine in MI repair.

At least two phase I clinical trials have demonstrated that transplantation of homogeneous heart-derived CSCs to patients is beneficial for heart function recovery [5-7]. However, the isolation of CSCs from heart tissue is potentially damaging and *in vitro* maintenance of cell populations is complex and time consuming. An improved understanding of the origin of CSCs should aid progress towards effective, viable treatment options. Presently, little is known concerning the origin of heart-derived CSCs. Heart resident CSCs may be either cells that have resided there from foetal life onwards or could come from the bone marrow or circulation [43]. An early report demonstrated that sex-mismatched heart transplantation patients possessed recipient-derived Y chromosomes in the nuclei of some cardiomyocytes, suggesting that bone marrow or circulation-derived stem cells had participated in the regenerative process [44]. We aimed to identify these alternative cells from bone marrow that possess properties of heart-derived CSCs.

We found that c-Kit^POS^/NKX2.5^POS^ cells within total BMSC populations display some properties of CSCs, being positive for NKX2.5 but negative for markers of adult cardiomyocytes, SMCs, and endothelial cells. Guo and colleagues proposed the existence of bone marrow-derived CSCs that express c-Kit and NKX2.5 [45]. Furthermore, these bone marrow-derived CSCs could repair injured hearts following transplantation to MI-model animals. Whether our current c-Kit^POS^/NKX2.5^POS^ BMSCs represent the same cell population as those identified by Guo and colleagues is interesting and warrants further study.

Notch1 signalling plays a pivotal role in the fate of heart-derived CSCs [17]. We found that c-Kit^POS^/NKX2.5^POS^ BMSCs were predisposed to Notch1 signalling-induced multilineage differentiation, with a preference for cardiac differentiation when compared with total BMSCs. Further clarification of the differences between c-Kit^POS^/NKX2.5^POS^ BMSCs and c-Kit^POS^ heart-derived CSCs may facilitate the clinical use of BMSCs.

Some limitations were present in our investigation. We provide *in vitro* evidence concerning Notch1 signalling in the differentiation of c-Kit^POS^/NKX2.5^POS^ BMSCs. However, the effects of Notch1 signalling activation in c-Kit^POS^/NKX2.5^POS^ BMSCs in an *in vivo* setting mimicking transplantation for repair of injured heart tissue post MI should be assessed in future studies. Additionally, more differentiation markers could be examined to better identify cell differentiation commitment and changes to cellular electrophysiological functions. Future studies should also focus on the optimal timing and degree of Notch signalling activation to promote cardiomyocyte differentiation of c-Kit^POS^/NKX2.5^POS^ BMSCs. The role of crosstalk between Notch and other signalling pathways should also be considered.

## Conclusions

Our study demonstrates the existence of c-Kit^POS^/NKX2.5^POS^ cells within total BMSC populations. These cells exhibit unique properties in colonic, proliferative, and multilineage differentiation potentials. c-Kit^POS^/NKX2.5^POS^ BMSCs are predisposed to Notch1-induced cell differentiation when compared with total BMSCs. Targeted activation of Notch1 signalling in c-Kit^POS^/NKX2.5^POS^ BMSCs leads to multilineage differentiation, suggesting that Notch1 signalling is a potential target for stem cell translational medicine research.

## Additional files

Additional file 1:
**is Table S1 presenting the primers used for PCR.** bp, base pairs. Additional file 2:
**is Table S2 presenting the primary antibodies.**
Additional file 3:
**is Figure S1 showing representative images of clones derived from cell of c-Kit** ^**POS**^ **/NKX2.5** ^**POS**^ **BMSCs.** c-Kit^POS^/NKX2.5^POS^ BMSCs were diluted into single-cell suspension and plated in six-well plates allowing it to form clones. Left panel, a small clone (the number of cells is <50), magnification: ×200; Right panel, two adjacent larger clones (the number of cells is >50), magnification: ×100. Additional file 4:
**is Figure S2 showing the growth curve of total BMSCs, c-Kit** ^**POS**^ **/NKX2.5** ^**POS**^ **BMSC and cardiac c-Kit** ^**POS**^ **CSC.** Total BMSCs, c-Kit^POS^/NKX2.5^POS^ BMSCs and cardiac c-Kit^POS^ CSCs were plated in six-well plates allowing cells to proliferate, and then total cell numbers were counted every day for consecutive 7 days. For isolation of cardiac CSCs, hearts form neonatal rat were used, and c-Kit^POS^ cells sorted from collagenase and trypsin-treated neonatal rat heart tissues by MACS were deemed c-Kit^POS^ CSCs. ****P* <0.001 versus other groups. Each cell line was repeated in three wells on every time point (*n* = 3). Additional file 5:
**is Figure S3 showing the adipogenic and osteogenic differentiation in total BMSCs and c-Kit** ^**POS**^ **/NKX2.5** ^**POS**^ **BMSCs.** (**A**) Total BMSCs and c-Kit^POS^/NKX2.5^POS^ BMSCs were induced to adipogenic and osteogenic differentiation using special inducing media for 3 weeks, and then cells were subjected to quantitative RT-PCR analysis of markers for adipocyte lineage (PPARγ2, FABP2) and osteoblast lineage (ALP, osteopontin). Data depicted as mean ± standard deviation from three independent experiments.**(B)** c-Kit^POS^/NKX2.5^POS^ BMSCs were infected with NICD-Ad and NC-Ad at multiplicity of infection = 100; cells without adenovirus infection was set as MOCK controls. Eight days post adenovirus infection, samples were used to quantitative RT-PCR analysis of marker for osteogenic differentiation (ALP) and adipogenic differentiation (PPARγ2) expression. Data depicted as mean ± standard deviation from three independent experiments. ***P* <0.01 versus other groups; ^###^ *P* <0.001 versus other groups. Additional file 6:
**is Figure S4 showing the effects of Jagged1 on Hes1 expression.** c-Kit^POS^/NKX2.5^POS^ BMSCs were seeded onto six-well plates. After cell recovery for a whole night, completed media containing recombinant Jagged1 (final concentration, 0, 1.25, 2.5 and 5.0 μg/ml) was added. After 8 days post Jagged1 treatment, samples were used to quantitative RT-PCR analysis Hes1 (target for judgement of Notch1 activation) expression. Data depicted as mean ± standard deviation from three independent experiments. ^***^ *P* <0.001 versus other groups.

## Abbreviations

BMC: bone marrow cell
BMSC: bone marrow mesenchymal stem cell
CSC: cardiac stem cell
cTnT: cardiac troponin T
DAPT: *N*-(2S-(3,5-difluorophenyl)acetyl)-l-alanyl-2-phenyl-1,1-dimethylethyl ester-glycine
DMEM: Dulbecco’s modified Eagle’s medium
FBS: foetal bovine serum
MACS: magnetic activated cell sorting
MI: myocardial infarction
NC-Ad: negative control-expressing adenovirus
NICD: notch receptor intracellular domain
NICD-Ad: NICD-expressing adenovirus
PBS: phosphate-buffered saline
SMC: smooth muscle cell
vWF: von Willebrand factor
