# Supplementary material for: Activation of Notch1 signalling promotes multi-lineage differentiation of c-KitPOS/NKX2.5POS bone marrow stem cells: implication in stem cell translational medicine
Source: Stem Cell Res Ther. 2015 May 9;6(1):91. doi: 10.1186/s13287-015-0085-2 (PMC4446115; doi:10.1186/s13287-015-0085-2)
Supplement: Additional file 1: — is Table S1 presenting the primers used for PCR. bp, base pairs. [file 13287_2015_85_MOESM1_ESM.pdf]

Additional file 1

Table S1. Primers used for PCR.

**Genes (ID)**	**Sequences for quantitative PCR (5'-3')**	**Product (bp)**
Hes1 (NM_024360.3)	gtgggtcctaacgcagtgtc<br>ctctcctaaagtccaagttcgtatt	165
NKX2.5 (NM_053651.1)	atggaaaggctcccactgt<br>aggcatcaggttaggtcaacaa	108
cTnT (NM_ 012676.1)	gactctgatcgaggctcact<br>attgcgaatacgctgctgtt	118
SM22α (Tagln) (NM_031549)	ctgtaatggctttgggcagt<br>ctcttatgctcctgggctttc	97
vWF (NM_053889.1)	acttcaccttcagtggcatct<br>agacagcatcagggtcatcag	111
β-actin (NM_031144)	cccatctatgagggttacgc<br>tttaatgtcacgcacgatttc	150
ALP (J03572.1)	gacggtgaacgggagaacg<br>cctcagaacagggtgcgtag	305
Osteopontin (NM_012881.2)	gaggagaaggcgcattacag<br>atggctttcattggagttgc	165
PPARγ2 (AF156666)	ccctggcaaagcatttgtat<br>actggcacccttgaaaaatg	222
FABP2 (NM_013068)	gtggtgaagaggaagcttgg<br>ccagaaatctctcggacagc	257
**Genes (ID)**	**Sequences for semi-quantitative PCR (5'-3')**	**Product (bp)**
c-Kit (NM_022264.1)	gaaagggaggccctaatgtc<br>cgtttgagctgtcacaggaa	259
NKX2.5 (NM_053651.1)	ttccagaaccgccgctacaag<br>ccgacgccaaagttcacgaag	323
α-MHC (NM_017239)	ggcacagaagatgctgacaa<br>ctgccccttggtgacatact	117
SM22α (Tagln) (NM_031549)	gaggactgtaatggctttgg<br>gccttccctttctaactgat	237
vWF (NM_053889.1)	cctacggcttgcactattca<br>ccacttcctcttccgacttac	301
β-actin (NM_031144)	tgcccatctatgagggttac<br>ctggaaggtggacagtgag	572
