# Supplementary material for: Activation of Notch1 signalling promotes multi-lineage differentiation of c-KitPOS/NKX2.5POS bone marrow stem cells: implication in stem cell translational medicine
Source: Stem Cell Res Ther. 2015 May 9;6(1):91. doi: 10.1186/s13287-015-0085-2 (PMC4446115; doi:10.1186/s13287-015-0085-2)
Supplement: Additional file 2: — is Table S2 presenting the primary antibodies. [file 13287_2015_85_MOESM2_ESM.pdf]

Additional file 2

Table S2. Primary antibodies.

**Antibody**	**Antibody**	**Application**	**Working dilution**
Rabbit anti-c-Kit	Santa Cruz Cat. #sc-168	Magnetic activated cell sorting, MACS	1:50
Goat anti-c-Kit	Santa Cruz Cat. #sc-1594	Immunofluorescence	1:50
Rabbit anti-NICD	Cell Signaling Cat. #4147S	Immunofluorescence	1:200
Rabbit anti-Hes1	Cell Signaling Cat. #11988S	Immunofluorescence	1:200
Goat anti-NKX2.5	Santa Cruz Cat. #sc-8697	Immunofluorescence	1:50
Rabbit anti-SM22α	Santa Cruz Cat. #sc-50446	Immunofluorescence	1:50
Mouse anti-SM-MHC	Santa Cruz Cat. #sc-6956	Immunofluorescence	1:50
Mouse anti-Sarcomeric actin	Sigma Cat. #1312	Immunofluorescence	1:200
Rabbit anti-vWF	Santa Cruz Cat. #sc-14014	Immunofluorescence	1:50
Rabbit anti-cTnT	Bioss Cat.#bs-2804	Immunofluorescence	1:100
Goat anti-Notch1	Santa Cruz Cat. Sc-6014	Flow cytometry	1:50
Rabbit anti-Notch2	Santa Cruz Cat. Sc-5545	Flow cytometry	1:50
Rabbit anti-Notch3	Santa Cruz Cat. #Sc-5593	Flow cytometry	1:50
Rabbit anti-Notch4	Santa Cruz Cat.#sc-5594	Flow cytometry	1:50
