# Supplementary material for: Activation of Notch1 signalling promotes multi-lineage differentiation of c-KitPOS/NKX2.5POS bone marrow stem cells: implication in stem cell translational medicine
Source: Stem Cell Res Ther. 2015 May 9;6(1):91. doi: 10.1186/s13287-015-0085-2 (PMC4446115; doi:10.1186/s13287-015-0085-2)
Supplement: Additional file 3: — is Figure S1 showing representative images of clones derived from cell of c-Kit POS /NKX2.5 POS BMSCs. c-KitPOS/NKX2.5POS BMSCs were diluted into single-cell suspension and plated in six-well plates allowing it to form clones. Left panel, a small clone (the number of cells is <50), magnification: ×200; Right panel, two adjacent larger clones (the number of cells is >50), magnification: ×100. [file 13287_2015_85_MOESM3_ESM.pdf]

Additional file 3

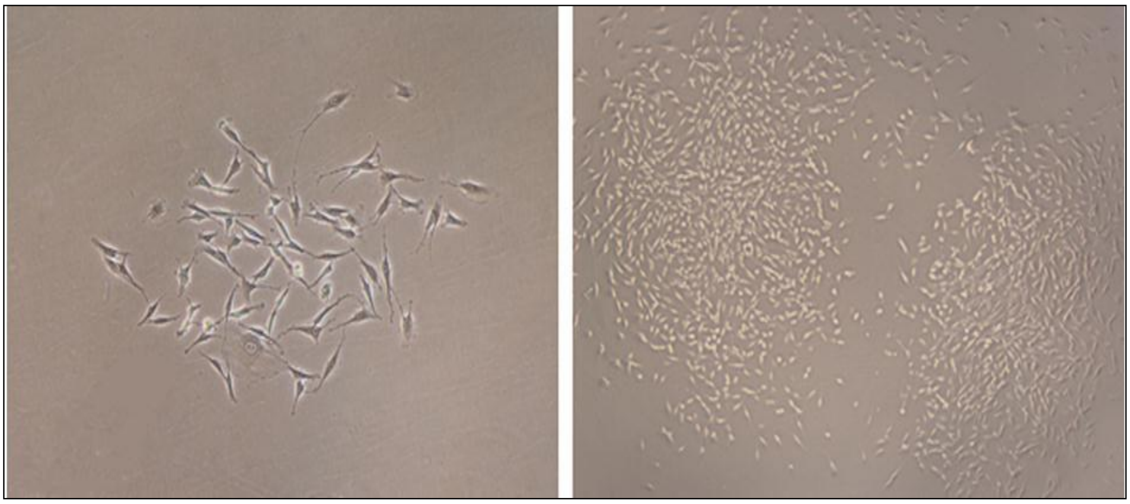

Figure S1. Representative images of clones derived from single cell of c-Kit^{POS}/NKX2.5^{POS} BMSCs. c-Kit^{POS}/NKX2.5^{POS} BMSCs were diluted into single cell suspension and plated in 6-well plates allowing it to form clones. Left panel, a small clone (the number of cells is <50), magnification: ×200; Right panel, two adjacent larger clones (the number of cells is >50), magnification: ×100.
