# Supplementary material for: Activation of Notch1 signalling promotes multi-lineage differentiation of c-KitPOS/NKX2.5POS bone marrow stem cells: implication in stem cell translational medicine
Source: Stem Cell Res Ther. 2015 May 9;6(1):91. doi: 10.1186/s13287-015-0085-2 (PMC4446115; doi:10.1186/s13287-015-0085-2)
Supplement: Additional file 4: — is Figure S2 showing the growth curve of total BMSCs, c-Kit POS /NKX2.5 POS BMSC and cardiac c-Kit POS CSC. Total BMSCs, c-KitPOS/NKX2.5POS BMSCs and cardiac c-KitPOS CSCs were plated in six-well plates allowing cells to proliferate, and then total cell numbers were counted every day for consecutive 7 days. For isolation of cardiac CSCs, hearts form neonatal rat were used, and c-KitPOS cells sorted from collagenase and trypsin-treated neonatal rat heart tissues by MACS were deemed c-KitPOS CSCs. ***P <0.001 versus other groups. Each cell line was repeated in three wells on every time point (n = 3). [file 13287_2015_85_MOESM4_ESM.pdf]

Additional file 4

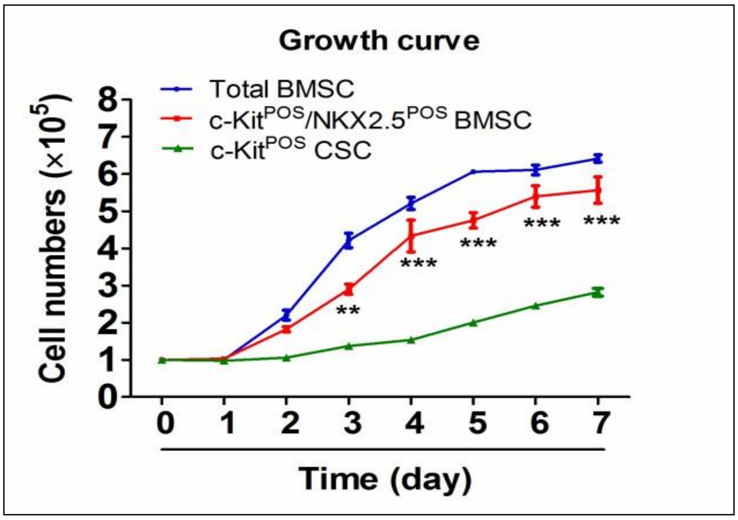

Figure S2. Growth curve of total BMSC, c-Kit^{POS}/NKX2.5^{POS} BMSC and cardiac c-Kit^{POS} CSC. Total BMSCs, c-Kit^{POS}/NKX2.5^{POS} BMSCs and cardiac c-Kit^{POS} CSC were aggregated and maintained in DMEM supplemented with 10% FBS, 100 U/mL LIF, 0.2 mmol/L glutathione, 2.5 U/mL EPO, and 10 ng/mL recombinant FGF-basic. 1×10^5 cells of total BMSCs, c-Kit^{POS}/NKX2.5^{POS} BMSCs and cardiac c-Kit^{POS} CSCs were plated in 6-well plates allowing cell to proliferate, and then total cells numbers were counted every day for consecutive 7 days. For isolation of cardiac CSCs, hearts form neonatal rat were used, and c-Kit^{POS} cells sorted from collagenase and trypsin-treated neonatal rat heart tissues by MACS were deemed as c-Kit^{POS} CSCs. The cardiac c-Kit^{POS} CSCs could form cardiosphere structures *in vivo*. ***P<0.001 *vs.* other groups. Each cell line was repeated 3 wells on every time points (n = 3).
