# Supplementary material for: Activation of Notch1 signalling promotes multi-lineage differentiation of c-KitPOS/NKX2.5POS bone marrow stem cells: implication in stem cell translational medicine
Source: Stem Cell Res Ther. 2015 May 9;6(1):91. doi: 10.1186/s13287-015-0085-2 (PMC4446115; doi:10.1186/s13287-015-0085-2)
Supplement: Additional file 5: — is Figure S3 showing the adipogenic and osteogenic differentiation in total BMSCs and c-Kit POS /NKX2.5 POS BMSCs. (A) Total BMSCs and c-KitPOS/NKX2.5POS BMSCs were induced to adipogenic and osteogenic differentiation using special inducing media for 3 weeks, and then cells were subjected to quantitative RT-PCR analysis of markers for adipocyte lineage (PPARγ2, FABP2) and osteoblast lineage (ALP, osteopontin). Data depicted as mean ± standard deviation from three independent experiments.(B) c-KitPOS/NKX2.5POS BMSCs were infected with NICD-Ad and NC-Ad at multiplicity of infection = 100; cells without adenovirus infection was set as MOCK controls. Eight days post adenovirus infection, samples were used to quantitative RT-PCR analysis of marker for osteogenic differentiation (ALP) and adipogenic differentiation (PPARγ2) expression. Data depicted as mean ± standard deviation from three independent experiments. **P <0.01 versus other groups; ### P <0.001 versus other groups. [file 13287_2015_85_MOESM5_ESM.pdf]

Additional file 5

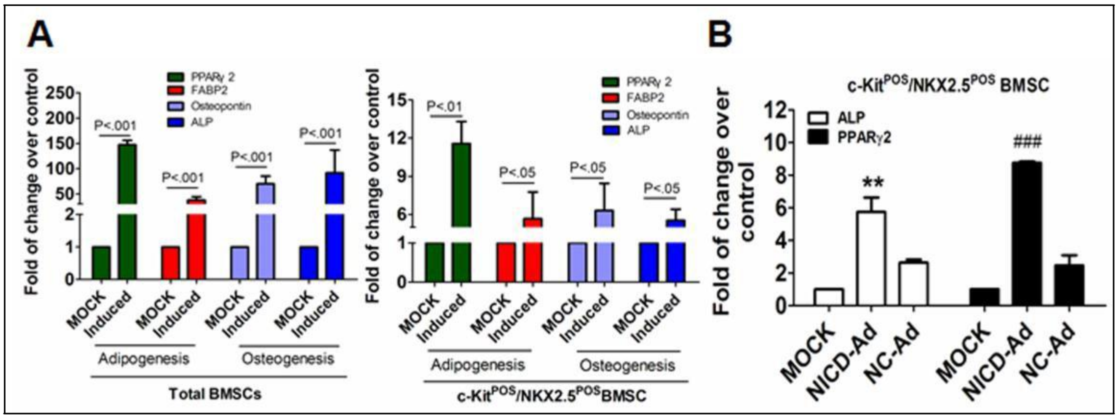

Figure S3. Adipogenic and osteogenic differentiation in total BMSCs and c-Kit^{POS}/NKX2.5^{POS} BMSCs. (A) Total BMSCs and c-Kit^{POS}/NKX2.5^{POS} BMSCs were seeded onto 6-well plates, then cells were induced to adipogenic and osteogenic differentiation using special inducing media (Cat:#RASMX-9002 & #RASMX-90031, Cyagen Biosciences, Guangzhou, China) for 3 weeks, then cells were subjected to quantitative RT-PCR analysis of markers for adipocyte lineage (PPARγ2, FABP2) and osteblast lineage (ALP, Osteopontin). Data were depicted as Mean ± SD from 3 independent experiments. **(B)** c-Kit^{POS}/NKX2.5^{POS} BMSCs were seeded onto 6-well plates, Notch1 intercellular domain (NICD) expression adenovirus (NICD-Ad) and negative control adenovirus (NC-Ad) were added at the MOI = 100, cells without adenovirus infection was set as MOCK controls. First times of media changing was carried out 24 hours post infection, then media were changed every 3 days. Eight days post adenovirus infection, samples were used to quantitative RT-PCR analysis of marker for osteogenic differentiation (ALP) and adipogenic differentiation (PPARγ2) expression. Data were depicted as Mean ± SD from 3 independent experiments. $^{**}P<0.01$ *vs*. other groups; $^{###}P<0.001$ *vs*. other groups.
