# Supplementary material for: Activation of Notch1 signalling promotes multi-lineage differentiation of c-KitPOS/NKX2.5POS bone marrow stem cells: implication in stem cell translational medicine
Source: Stem Cell Res Ther. 2015 May 9;6(1):91. doi: 10.1186/s13287-015-0085-2 (PMC4446115; doi:10.1186/s13287-015-0085-2)
Supplement: Additional file 6: — is Figure S4 showing the effects of Jagged1 on Hes1 expression. c-KitPOS/NKX2.5POS BMSCs were seeded onto six-well plates. After cell recovery for a whole night, completed media containing recombinant Jagged1 (final concentration, 0, 1.25, 2.5 and 5.0 μg/ml) was added. After 8 days post Jagged1 treatment, samples were used to quantitative RT-PCR analysis Hes1 (target for judgement of Notch1 activation) expression. Data depicted as mean ± standard deviation from three independent experiments. *** P <0.001 versus other groups. [file 13287_2015_85_MOESM6_ESM.pdf]

Additional file 6

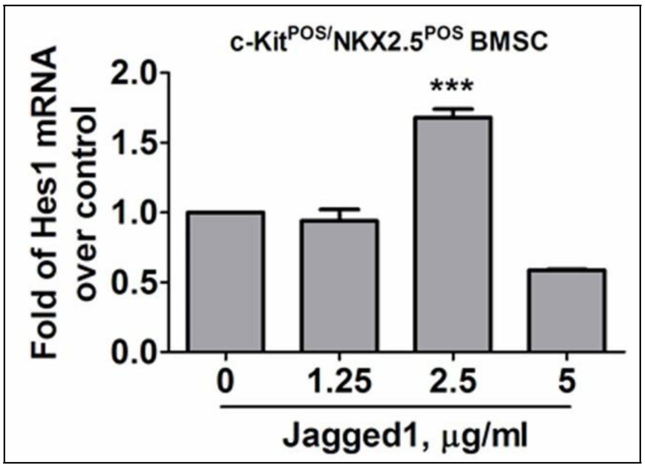

Figure S4. Effects of Jagged1 on Hes1 expression. c-Kit^{POS}/NKX2.5^{POS} BMSCs were seeded onto 6-well plates. After cell recovery for whole night, completed media containing recombinant Jagged1 (final concentration, 0, 1.25, 2.5 and 5.0 μg/ml) was added. After 8 days post Jagged1 treatment, samples were used to quantitative RT-PCR analysis Hes1 (target for judgment of Notch1 activation) expression. Data were depicted as Mean ± SD from 3 independent experiments. ****P*<0.001 *vs*. other groups.
